# Nurse Professional Competence as a Mediator Between Structural Empowerment and Person-Centered Climate in Hospital Settings: A Cross-Sectional Observational Study

**DOI:** 10.1155/jonm/2078705

**Published:** 2025-09-29

**Authors:** Lijuan Xu, Kewen Zhu, Annika Nilsson, Maria Engström

**Affiliations:** ^1^Department of Nursing Science, Medicine College, Lishui University, Lishui, China; ^2^Department of Caring Science, Faculty of Health and Occupational Studies, University of Gävle, Gävle, Sweden

**Keywords:** nurse competence, nursing, person-centered care, person-centered climate, structural empowerment

## Abstract

**Background:**

The work-related empowerment of nurses is essential for enhancing person-centered care and climate. However, research on more complex relationships that consider mediation remains sparse.

**Objective:**

This study aimed to investigate the relationship between nurse-rated structural empowerment and a person-centered climate, as well as the mediating role of nurse professional competence.

**Design:**

A cross-sectional survey study.

**Settings and Participants:**

This study was conducted among 2172 nurses, at three general hospitals in China, between April 2023 and October 2023.

**Methods:**

The Conditions of Work Effectiveness Questionnaire, which measured structural empowerment, the Nurse Professional Competence Scale, and the Person-Centered Climate Questionnaire-Staff Version were employed to collect data. The PROCESS macro (Model 4) and bootstrapping tests were used to examine the relationships and mediation effects.

**Results:**

The results showed that nurse-rated structural empowerment was significantly positively related to the person-centered climate and professional competence of nurses. Nurse professional competence, in turn, was significantly positively related to person-centered climate. Furthermore, the nurse professional competence significantly mediated the relationship between structural empowerment and a person-centered climate. The model explained 49.9% of the variance in person-centered climates.

**Conclusions:**

Structural empowerment and nurses professional competence are both essential for fostering a person-centered climate in hospital wards. Professional competence mediates the relationship between structural empowerment and a person-centered climate.

**Implications for Nursing Management:**

Nurse managers and policymakers should promote empowering work environments by ensuring access to information, support, resources, and opportunities for professional development. Enhancing both formal empowerment (e.g., recognizing and elevating the role of nurses within the organization) and informal empowerment (e.g., supporting meaningful professional networks) can help nurses strengthen their competence and contribute to a more person-centered climate at the unit level.

## 1. Introduction

Person-centered care and climate are the fundamental aspects of care quality, and their significance has gained global recognition, including increasing attention in China in recent years [1]. In the context of care quality and staff performance, structural empowerment theory [2] highlights the importance of providing staff with access to empowering conditions. Previous research has established links between structural empowerment and various indicators of care quality, including staff-rated care quality [3–5], patient satisfaction [6], and staff perceptions of person-centered care and climate [7]. Additionally, structural empowerment has been associated with staff well-being [8] and nurse professional competence [9]. Nurse competence, in turn, has been shown to relate to person-centered care [10–12]. However, the complex interrelationships among these factors, particularly how structural empowerment influences person-centered climate, have not been fully explored. In particular, the potential mediating role of nurse professional competence in this relationship warrants further investigation.

As a theoretical framework in the study, we used both the person-centered care framework [13, 14] and Kanter's theory of structural empowerment [2]. Person-centered care is described in various ways, but common themes are consistently emphasized. Some definitions focus on the patient's narrative, highlighting their health experiences, strengths, and personal goals, along with a collaborative partnership with healthcare staff and the documentation of shared goals in medical records, for example, Moore et al. [15]. Others also consider broader contextual factors and the care environment. For example, McCormack and McCance outline five key domains in their person-centered care framework: the macro context, prerequisites, care environment, person-centered processes, and expected outcomes [13, 14]. In their framework [13, 14], the macro context refers to organizational factors that influence the development of a person-centered culture within healthcare institutions. Prerequisites are the attributes and competencies of staff (e.g., professional competence), while the care environment encompasses the clinical context, including access to supportive organizational structures (such as structural empowerment) and the physical setting. Person-centered processes involve the direct interactions between staff and patients in the delivery of care. Finally, expected outcomes are the measurable results of effective person-centered care, such as patient satisfaction.

The framework recognizes that relationships exist both within and across these domains, highlighting the complexity and interdependence of the elements that support person-centered care [13].

In the present study, the outcome of interest was person-centered climate, defined as a care environment, both physical and psychosocial, that treats the patient as a person and centers their needs and expectations in the delivery of care [16]. This includes the tangible environment, such as a homelike and peaceful atmosphere with aesthetically pleasing surroundings (cf. the practice environment domain in the person-centered care framework), as well as the quality of care and interactions between staff and patients (cf. the person-centered processes domain) [16].

Kanter's theory of structural empowerment was used in this study to examine hospital nurses' access to organizational conditions that support and empower their work. These conditions correspond to the “practice environment” domain in the person-centered care framework. According to Kanter's theory [2], structural empowerment consists of four key components: *staff access to information* about work and the organization; access to *resources*, such as time, people, and equipment to perform work; access to *support*, such as the feedback from managers and colleagues; and *opportunities* to learn, develop, and achievement. Additionally, the theory highlights two types of power that facilitate the access to these empowering structures: *formal power* (a central and visible work within the organization) and *informal power* (networks within and outside the hospital/unit that ease work). According to the theory, strong access to these empowering conditions enhances both staff wellbeing and performance.

Based on Kanter's theory of structural empowerment, Laschinger et al. [17] developed an empowerment framework tailored to nursing work settings. This framework suggests that access to empowering structures leads to psychological empowerment, which includes four cognitive dimensions: a sense of meaning, competence, self-determination, and impact [18]. Nurses with higher psychological empowerment are, in turn, more likely to facilitate patient access to empowering structures. Structural empowerment is, therefore, linked to nurse competence and the delivery of person-centered care.

Several factors have been identified as important for person-centered care and climate, including organizational conditions such as staff structural empowerment [7], staff competence [10–12], educational level [19, 20], clinical experience [20], and being a nurse specialist or not [20]. These elements align with the *practice environment* and *prerequisites* domains in the person-centered care framework. Other demographic and work-related factors shown in research to be associated with staff-rated person-centered care or climate include work shift (day vs. night) [21], age (statistically significant in one study [22] but nonsignificant in another [21]), sex [19, 23], marital status [20, 24], monthly income [23], employment [23], and technical title [19]. A metasynthesis of nurses' experiences in implementing person-centered care indicated that both organizational conditions and professional competence are essential prerequisites for person-centered care [25]. Similarly, a literature review on person-centered care in China [26] highlighted influencing factors such as a supportive sociocultural context, community coordination, and a shared understanding between staff and older adults.

Extensive research has established a strong relationship between structural empowerment and staff well-being [5, 9, 27–29]. There is also evidence linking structural empowerment to staff performance, including quality of care as rated by staff [3, 4, 30], staff-perceived person-centered care and climate [7, 31], and patient satisfaction with care [6]. Additionally, structural empowerment has been associated with professional competence [9, 32]. Furthermore, higher scores in the practice environment, which encompasses elements similar to structural empowerment, have been linked to both higher nurse competence and improved quality of care, as reported in a review of nursing competence [33, 34]. In turn, nurse competence has consistently been associated with person-centered care in previous studies [10–12, 35].

In summary, previous studies have identified associations between structural empowerment and person-centered care and climate, structural empowerment and nurses' professional competence, and nurse professional competence and person-centered care. These relationships align with both the person-centered care framework and the theory of structural empowerment. However, a gap remains in the literature regarding the direct and indirect effects involved in these relationships. One study has explored the direct and indirect effects of nurse competence on person-centered care, reporting both a direct effect and an indirect effect mediated by self-efficacy and empathy [35]. Yet the mediating role of nurse professional competence in the relationship between staff access to empowering structures and person-centered care has not been thoroughly examined. Moreover, in countries such as China and South Korea, most research on person-centered care has focused on elderly care services, particularly in dementia care, with limited attention to hospital-based nursing contexts [35, 36].

Thus, the present study investigated the relationship between structural empowerment and a person-centered climate and the mediating role of nurse professional competence among Chinese hospital nurses based on (1) the person-centered care framework where the domains prerequisites and practice environment (including aspects, such as supportive organizational conditions) are supposed to influence person-centered processes, (2) the empowerment framework where structural empowerment is supposed to influence cognitive functions (here measured as professional competence), and (3) earlier research, two hypotheses were formulated.

**Figure 1:**
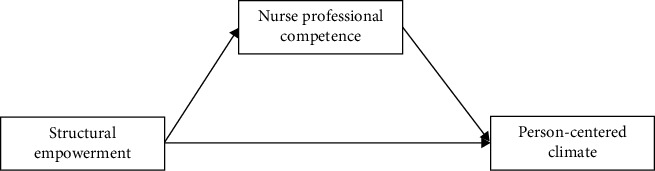
Hypothesized framework.

• Hypothesis 1: there is a positive relationship between structural empowerment and a person-centered climate.
• Hypothesis 2: the relationship between structural empowerment and a person-centered climate is positively mediated by nurse professional competence (Figure 1 describes the hypothesized relationships and the model to be tested).

## 2. Methods

### 2.1. Study Design

This study used a cross-sectional correlational design to examine the relationships between structural empowerment, nurse professional competence, and a person-centered climate among Chinese general hospital nurses.

### 2.2. Participants

A convenience sample of registered nurses working in three general hospitals, which were also teaching hospitals, in a city in Southeast China was asked to participate during April and October 2023. To enhance the representativeness of our sample, we purposively selected three hospitals that serve as the primary tertiary care institutions within the study region. The involved departments in the three hospitals included different specialties, such as medical nursing, surgical nursing, obstetrics and gynecology nursing, pediatrics nursing, acute and critical care nursing, rehabilitation nursing, geriatric nursing, and psychological nursing. The total number of eligible participants, registered nurses from all departments of each general hospital, was 2586. Ultimately, 2172 nurses from 121 departments participated in this study. As per the inclusion criteria, individuals who were registered nurses working in one of three hospitals were included in the study. Registered nurses who were on sick leave, maternity leave, or educational leave at the time of the study were excluded.

### 2.3. Data Collection

Prior to data collection, the first author contacted the dean of the nursing department at each participating hospital and obtained permission to conduct the study. From the deans, a list of units was obtained, including the number of registered nurses per unit and the contact information for each unit's head nurse. The head nurses were then contacted individually, and appointments were scheduled for data collection. The head nurses distributed the questionnaires to all registered nurses in their respective units. Participation was anonymous, and participants were assured that their data would be securely stored and inaccessible to unauthorized individuals. They were also informed that all quantitative data would be reported at the group level only. Two reminders were sent to encourage participation. A total of 2172 completed questionnaires were returned, resulting in a response rate of 83.9%.

### 2.4. Measurement

Structural empowerment was measured using the Conditions of Work Effectiveness Questionnaire II (CWEQ-II) [17], a widely used and validated instrument among healthcare workers across various settings and countries. The CWEQ-II consists of 19 items across six subscales: opportunity (3 items), information (3 items), support (3 items), resources (3 items), formal power (3 items), and informal power (4 items). Each item is rated on a 5-point Likert scale, ranging from 1 (never) to 5 (very often). For scoring, a mean score is calculated for each subscale, and these are then summed to produce a total empowerment score. Higher scores indicate higher levels of structural empowerment, with total scores categorized as follows: 6–13: low empowerment; 14–22: moderate empowerment; and 23–30: high empowerment [17]. The Chinese version of the CWEQ-II has been validated in samples of Chinese nurses. Both exploratory and confirmatory factor analyses demonstrated acceptable construct validity. The instrument also showed strong internal consistency, with a Cronbach's alpha (α) of 0.94 for the total scale, and subscale alphas ranging from 0.76 to 0.91 [37]. Values of α above 0.70 are considered acceptable, while values of 0.80 or higher are preferred for research use [38, 39].

Nurse professional competence was measured using the Nurse Professional Competence Scale-Short Form (NPC-SF) [40, 41]. The scale was developed based on the competence requirements outlined by the World Health Organization and the Swedish Society of Nursing, and it has been used and validated in multiple countries [42]. The NPC-SF consists of 35 items across six competence domains: Nursing care (5 items); Value-based nursing care (5 items); Medical and technical care (6 items); Care pedagogics (5 items); Documentation and administration of nursing care (8 items); and Development, leadership, and organization of nursing care (6 items). Responses are rated on a 7-point Likert scale ranging from 1 (*very low*) to 7 (*very high*). Total and subscale scores are calculated as raw scores, divided by the highest possible score for each, and multiplied by 100 to produce a percentage score.

The Chinese version of the NPC-SF demonstrated strong psychometric properties. Confirmatory factor analysis and known-groups validity confirmed the use of the same six-factor structure as the original Swedish version. The scale showed excellent reliability, with a Cronbach's alpha (α) of 0.97 for the total score among Chinese nursing students at the time of graduation [43].

Person-centered climate was assessed using the Person-Centered Climate Questionnaire-Staff Version (PCQ-S) [16]. This instrument has been translated into Chinese, English [44], and Norwegian [45] and is widely used and validated in both hospital and nursing home settings internationally. The Chinese version contains 14 items grouped into three factors: Climate of safety (5 items); Everydayness (5 items); and Community (4 items). Each item is rated on a 6-point scale from 0 (*completely disagree*) to 5 (*completely agree*). Higher scores indicate a more person-centered climate. In the validation of the Chinese version, both exploratory and confirmatory factor analyses demonstrated good construct validity. The scale showed strong reliability, with a Cronbach's alpha (α) of 0.94 for the total score [46].

Demographic information, including age [19, 20], sex [19, 23], marital status [20, 24], educational level [19, 20], clinical experience [20], monthly income [23], nurse specialist [20], employment [20], technical title [19], and night or day shifts [19], have all shown relationships with nurse-rated person-centered care in previous studies. The above variables were considered as covariates based on the previous literature that identified their potential influence on the person-centered climate to examine the relationships more accurately between the study variables. The covariates were included in the mediation model if the *p* values for the outcome in bivariate correlation analysis, *t*-test, or analysis of variance were less than 0.1.

### 2.5. Ethical Considerations

Before the study started, an application to ×× University's Medical Ethics Board (no. 2023YR020) and permission from the nursing department committee were obtained. The study adhered to the principles of the Declaration of Helsinki [47] and Ethical Review of Life Sciences and Medical Research Involving Humans in China [48]. All the participants received written information outlining the purpose and procedures of the study. They were informed of their right to participate voluntarily and their ability to decline or withdraw at any time without consequence. The data collection was anonymous, and participants were assured that all data would be securely stored to prevent unauthorized access. Additionally, they were informed that all quantitative data would be reported at the group level, ensuring individual confidentiality.

### 2.6. Data Analysis

Among the study variables, 48 cases had missing values ranging from 1 to 19 items on structural empowerment scale, 53 cases had missing values ranging from 1 to 35 items on the Nurse Professional Competence scale, and 64 cases had missing values ranging from 1 to 14 items on the person-centered climate questionnaire. Cases with 50% or more missing items within a given scale were excluded from the analysis. For cases with less than 50% missing items on a scale, missing values were imputed. Missing data for covariates ranged from 7 to 100 cases. Thus, 1928 cases were included in the final analysis to test hypothesized relationships.

A data analysis was conducted using IBM SPSS Statistics (Version 28.0) and PROCESS (Version 4.0) Model 4 [49]. To test the relationships between the sociodemographic characteristics and person-centered care climate, *t*-test, one-way analysis of variance, and Pearson's correlation coefficient were used. Those with *p* values < 0.1 were included as covariates in the models of multiple relationships: sex (male and female), marital status (married couple and others), nurse specialist (yes or no), and night shift (yes or no). To test multiple relationships and mediation, we used linear regression, PROCESS macro Model 4.

Model 4 is used for simple mediation analysis involving one mediator [49]. In the present study, both the direct and indirect effects of structural empowerment on person-centered climate were tested, with nurse professional competence as the mediator. To estimate the indirect effect and calculate confidence intervals (CIs), a bootstrapping procedure was performed using 5000 bootstrap samples and 95% CIs. If zero was not included within the 95% CIs, the effect was considered statistically significant. Bootstrapped CIs are preferred because they better accommodate the non-normal sampling distribution of the indirect effect (ab), resulting in more accurate inferences than the traditional normal theory approach [49]. Additionally, bootstrapping generally provides higher statistical power [49].

## 3. Results

### 3.1. Demographic Characteristics of Participants

A total of 2172 nurses were included in this study. The mean age of the participants was 30.3 years (standard deviation [SD] = 7.7), with an average of 9.5 years (SD = 8.0) of clinical experience. Most were female (*n* = 2069) and had a bachelor's degree (*n* = 1463). The details of participant characteristics and mean values for the different instruments are presented in Tables 1 and 2.

### 3.2. Descriptive Statistics and Relationships Between Study Variables

The means, SD, and correlations among the variables are shown in Table 2. The nurses' perception of structural empowerment was 21.3 (SD = 4.9), indicating a moderate level. The mean values of perceived nurse professional competence and person-centered climate were 89.3 (SD = 10.8) and 56.2 (SD = 12.3), respectively. The results showed that structural empowerment was positively related to nurse professional competence (*r* = 0.54, *p* < 0.001) and person-centered climate (*r* = 0.70, *p* < 0.001), supporting the hypothesis that structural empowerment is positively related to nurse professional competence and person-centered climate. In addition, nurse professional competence was positively related to a person-centered climate (*r* = 0.55, *p* < 0.001).

### 3.3. Mediation Effect

**Figure 2:**
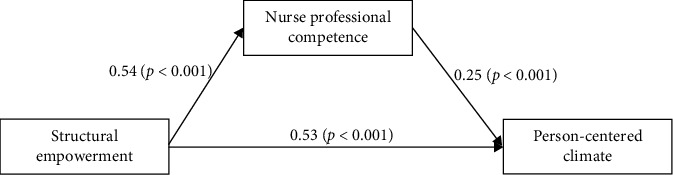
The mediation model, standardized coefficients. Note: Controlling sex, marital status, specialist, and night shift.

The results showed that the mediation model explained 49.9% of the variance in person-centered climate, indicating that nearly half of the variation in person-centered climate among nurses can be attributed to the combined effects of structural empowerment and nurse professional competence. The total effect of structural empowerment on person-centered climate was 0.67 (*p* < 0.001). The standardized direct effect of structural empowerment on person-centered climate was 0.53 (*p* < 0.001), while the standardized indirect effect through nurse professional competence was 0.13 (95% CI: 0.104–0.167).  Table 3 and Figure 2 illustrate the mediation model along with the direct and indirect effects. Since the CI for the indirect effect does not include zero, the mediation effect is statistically significant, supporting Hypothesis 2. These results suggest that part of the impact of structural empowerment on person-centered climate is mediated by nurses' professional competence, underscoring the importance of competence development in improving the work environment.

## 4. Discussion

This study contributes new insights by identifying nurse professional competence as a mediator in the relationship between empowering structures and person-centered climates. Consistent with our hypotheses, we found that structural empowerment is positively associated with a person-centered climate, and this relationship is mediated by nurse professional competence.

The positive relationship between structural empowerment and person-centered climate is consistent with previous research by Silen et al. [7] on person-centered climate and person-centered care by Alhalal et al. [31] and supports Kanter's [2] theory of structural empowerment. According to Kanter's theory of structural empowerment, organizational conditions that provide staff with access to information, resources, support, opportunities, and both formal and informal empowerment improve staff well-being and work quality. While previous reviews have shown the importance of these empowering structures for nursing care quality [50], research specifically linking them to person-centered care is limited. In our study, participant characteristics, such as sex, marital status, specialist nurse vs. not, and night shift vs. not, were statistically significant in univariate and bivariate analyses but nonsignificant in the multiple regression analysis. This is also in line with Kanter's [2] theory that empowering organizational conditions is more important than personal characteristics for work quality measured here as a person-centered climate. Our study also highlights the relationship between structural empowerment and nurse professional competence (c.f. research by Högstedt et al.) [9]. Furthermore, the results align with McCance's and McCormack's [14] person-centered care framework, showing that both the “practice environment” (structural empowerment) and “prerequisites” (nurse competence) are important for creating a person-centered climate.

Our results revealed that the professional competence of nurses mediated the relationship between structural empowerment and person-centered climate. These findings support the person-centered care framework [14, 51] that emphasizes staff professional competence linked to person-centered care. These findings are consistent with those of previous research that found structural empowerment to be related to the nurse-rated professional competence [9]. Also, in the research by Pakkonen et al. [11, 12], it was observed that improving the nursing competence in person-centered care positively influenced the person-centered climate and nursing care culture.

Our results demonstrate that structural empowerment is related to a person-centered climate, with this relationship mediated by nurse professional competence. Indeed, approximately 50% of the variance in person-centered climate remains unexplained, pointing to the involvement of additional mediating or moderating variables.

Additionally, we did not measure factors from the “macro context” domain of the person-centered care framework, such as policy documents and strategic leadership, which could also influence person-centered cultures and thus affect the results. Future research may consider incorporating these “macro context” factors from the person-centered care framework into extended or moderated mediation models to better capture the complex mechanisms underlying person-centered care process.

The values of nurse competence (mean: 89), person-centered climate (mean: 56), and structural empowerment (mean: 21) indicated higher values compared with previous studies of nurses (nursing competence mean of 82–87) for the different competence areas [52], person-centered climate mean of 53 [46], and structural empowerment mean of 19.6 [36]. The relatively high mean scores in both structural empowerment and professional competence observed in this study may be partially attributed to specific cultural and contextual factors within the Chinese healthcare system.

It is important to note that nurse professional competence in this study was assessed using self-rated measures, which reflect perceived rather than objectively observed competence. Nurse competence levels may also vary between countries depending on whether competence development is mandatory. For example, in China, hospital nurses are required to participate in training activities every month. National mandates for standardized training programs and continuing professional development—often tightly linked to licensure renewal and career advancement—have contributed to a uniformly high level of baseline competence among hospital nurses. Additionally, the hierarchical organizational structure typical of public hospitals in China may paradoxically contribute to perceived empowerment, as clearly defined roles, responsibilities, and leadership pathways can facilitate decision-making processes and participation in institutional governance among nurses. These cultural-contextual dynamics warrant further exploration in cross-cultural research on empowerment and competence in person-centered care environments.

### 4.1. Implications for Clinical Practice

Hospital organizations, nursing managers, and policymakers should ensure that staff have access to empowering structures and that nurses possess strong professional competence to provide high-quality care. To support this, hospitals must regularly assess staff access to these structures and address any shortcomings. Additionally, personalized career development plans for nurses are crucial for enhancing professional competence and promoting person-centered care. Laschinger [53] suggests that strategies to foster structural empowerment include establishing clear communication channels, offering visible recognition, ensuring adequate resources and supplies, facilitating advanced education, elevating the nursing role within organizational goals, and promoting interdisciplinary networking opportunities.

Zhang et al. emphasized that a supportive work environment, along with a fair performance incentive system and positive interpersonal relationships, is essential to improve nurses' work engagement, optimize performance, and enhance professional competence [54]. A systematic scoping review [11] found that continuing education interventions in person-centered care effectively strengthen nurses' competence in this area, leading to higher job satisfaction and better quality of care. Educational programs using narratives combined with reflective strategies, such as discussion groups, can cultivate nurses' professional competencies linked to person-centered care [10, 12, 55].

Thus, to cultivate a person-centered climate in hospital settings, it is essential for managers to implement the following targeted strategies. One key intervention is the adoption of transformational leadership, which encourages open communication and a shared vision for person-centered care. Mentoring programs can further support nurses' professional development by providing individualized guidance and strengthening their confidence in delivering empathetic care. Moreover, peer networks allow nurses to share experiences and coping strategies, contributing to a supportive organizational culture. Narrative-based training, such as storytelling and reflective writing workshops, can deepen staff understanding of patients' lived experiences, enhancing their capacity for compassionate care. However, this approach requires further investigation in future studies.

### 4.2. Limitations

It is worth noting that this study used a cross-sectional design, which makes it challenging to determine cause-and-effect relationships. Nevertheless, the links with existing theory and frameworks support these findings; however, longitudinal studies are needed in the future to validate the relationships among these variables. This study used self-reported measurements by nurses, which could have introduced a social desirability bias. For example, self-perceived competence may not always align with actual performance or objectively observed competence. Additionally, the patient ratings for person-centered climates could be used in future studies. Nevertheless, using validated instruments with high Cronbach's alpha values for the sample is a strength. Finally, the nurses were selected from three general hospitals in one region of China, which limits its generalizability to all Chinese nurses in, for example, teaching and specialized hospitals. A strength of the study is that the response rate was quite high. As there are discrepancies in the management model of different hospitals, the results could be affected by organizational cultures. Future studies should explore potential predictors and mediators beyond nurse professional competence. Attributes from all domains of the person-centered care framework could be considered.

## 5. Conclusions

The present study highlights the relationship between structural empowerment and a person-centered climate, mediated by nurse professional competence. The findings suggest that empowering work environments not only directly foster a person-centered climate but also do so indirectly by enhancing nurses' perceived competence.

Following Kanter's theory of structural empowerment, organizational conditions, such as good access to information about work, support, resources, opportunities to develop, formal (a visible and central job) and informal empowerment (networks within and outside the organization that ease work), are important for fostering a person-centered climate. Moreover, this mediating effect highlights the critical role of competence-building initiatives as an integral component of empowerment strategies. These findings offer valuable insights for hospital administrators, nurse leaders, and policymakers in designing targeted interventions that effectively align structural empowerment with professional development efforts, thereby fostering a more person-centered approach in clinical practice.

## Figures and Tables

**Table 1:** Characteristics of the participants and differences in nurse-rated person-centered climate (*N* = 2172).

Variables		*n* (%)	Mean (SD)	^a^ *p*
Gender	Female	2069 (95.3%)	53.3 (13.6)	**0.045**
	Male	88 (4.1%)	56.3 (12.2)	

Marital status	Single/divorced/widow (er)	1066 (49.1%)	55.6 (12.3)	0.061
	Married	1075 (49.5%)	56.6 (12.4)	

Education	Diploma	47 (2.2%)	58.4 (10.1)	0.399
	Associate degree	620 (28.5%)	56.0 (12.2)	
	Bachelor's degree	1463 (67.4%)	56.2 (12.5)	
	Master's degree	4 (0.2%)	49.0 (5.8)	

Technical title	None (without junior test)	679 (31.3%)	56.0 (12.1)	0.257
	Junior	687 (31.6%)	55.7 (12.7)	
	Intermediate	558 (25.7%)	56.3 (12.6)	
	Associate senior	197 (9.1%)	57.9 (11.1)	
	Senior	32 (1.5%)	56.0 (10.0)	

Nurse specialist	Yes	151 (7.0%)	59.0 (11.0)	**0.003**
	No	1919 (88.4%)	56.0 (12.3)	

Employment	Staff within establishment	1041 (47.9%)	56.7 (12.2)	0.145
	Contract staff	1056 (48.6%)	55.6 (12.5)	
	Others	43 (2%)	55.4 (11.0)	

Night shift	None	638 (29.4%)	57.2 (11.8)	**0.031**
	1-2 times/week	938 (43.2%)	55.7 (12.6)	
	3 times or more/week	528 (24.3%)	55.5 (12.4)	

*Note:* Bold text/figures indicate statistically significant results; when the sum does not add up to *n* = 2172, there are internal missing data.

^a^Independent *t*-test or ANOVA test.

**Table 2:** Descriptive statistics and Pearson correlation matrix of the study variables (*N* = 2172).

Variables	M	SD	Correlation					
			1	2	3	4	5	6
1. Age (years)	30.3	7.7	—					
2. Clinical experience (years)	9.5	8.0	0.96^∗∗∗^	—				
3. Monthly income (yuan)	5646.3	1697.2	0.44^∗∗∗^	0.43^∗∗∗^	—			
4. Structural empowerment	21.3	4.9	0.10^∗∗∗^	0.10^∗∗∗^	0.04	—		
5. Nurse professional competence	89.3	10.8	0.15^∗∗∗^	0.15^∗∗∗^	0.10^∗∗∗^	0.54^∗∗∗^	—	
6. Person-centered climate	56.2	12.3	0.04	0.04	−0.01	0.70^∗∗∗^	0.55^∗∗∗^	—

*Note:* The possible range of the scales is structural empowerment 6–30 (6–13: designate low, 14–22: moderate, and 23–30: high levels), nurse professional competence 14–100, and person-centered climate 0–70.

^∗∗∗^ *p* < 0.001.

**Table 3:** The mediation model, the effects, and 95% confidence interval (CI) (*N* = 1928).

Model pathways	Estimated	*p*	
Standardized direct effect			
Structural empowerment ⟶ nurse professional competence	0.54	< 0.001	
Nurse professional competence ⟶ person-centered climate	0.25	< 0.001	
Structural empowerment ⟶ person-centered climate	0.53	< 0.001	
Sex	0.02	0.171	
Marital status	−0.03	0.059	
Nurse specialist	−0.01	0.621	
Night shifts	−0.001	0.942	

**Standardized indirect effect**	**Estimated**	**95% CI**	
		**Lower**	**Upper**

Structural empowerment ⟶ person-centered climate	0.13	0.104	0.167

## Data Availability

The data that support the findings of this study are available on request from the corresponding author.
